# Effects of endothelial cell proliferation and migration rates in a computational model of sprouting angiogenesis

**DOI:** 10.1038/srep36992

**Published:** 2016-11-14

**Authors:** Kerri-Ann Norton, Aleksander S. Popel

**Affiliations:** 1 Department of Biomedical Engineering, School of Medicine, Johns Hopkins University Baltimore, Maryland 21205, USA.

## Abstract

Angiogenesis, the recruitment of new blood vessels, is a critical process for the growth, expansion, and metastatic dissemination of developing tumors. Three types of cells make up the new vasculature: tip cells, which migrate in response to gradients of vascular endothelial growth factor (VEGF), stalk cells, which proliferate and extend the vessels, and phalanx cells, which are quiescent and support the sprout. In this study we examine the contribution of tip cell migration rate and stalk cell proliferation rate on the formation of new vasculature. We calculate several vascular metrics, such as the number of vascular bifurcations per unit volume, vascular segment length per unit volume, and vascular tortuosity. These measurements predict that proliferation rate has a greater effect on the spread and extent of vascular growth compared to migration rate. Together, these findings provide strong implications for designing anti-angiogenic therapies that may differentially target endothelial cell proliferation and migration. Computational models can be used to predict optimal anti-angiogenic therapies in combination with other therapeutics to improve outcome.

Angiogenesis, the formation of new blood vasculature, is one of the hallmarks of cancer^1^,^2^ that is necessary for the transition from a contained tumor to invasive disease that eventually leads to metastasis. Initially, the lack of oxygen and nutrients prevents the growth of tumors in excess of 1–2 mm in diameter. However, in an event known as the ‘angiogenic switch’, populations of cells within the tumor are able to uncouple the regulation of pro-angiogenic factors and initiate neovascularization^3^. Notably, these cells release vascular endothelial growth factor A (VEGF-A or VEGF for brevity), a primary factor necessary for the initiation of sprouting angiogenesis^4^. VEGF promotes angiogenesis by binding to VEGF receptors VEGFR1 and VEGFR2 and co-receptors neuropilins-1 (NRP1) and −2 (NRP2) and is known to play a role in endothelial cell survival, proliferation, and migration^5^. Angiogenesis is initiated by the degradation of the extracellular matrix by matrix metalloproteinases, which clears a path for the developing sprout and releases proteases^6^. The developing sprout extends towards a VEGF gradient but there are several VEGF isoforms which have different affinities to VEGF receptors and binding to heparan sulfate proteoglycans resulting in distinct vascular architectures^7^.

The first step in angiogenesis occurs by the formation of a new sprout, off of the existing vasculature, mediated by tip cell migration and stalk cell proliferation. VEGF causes the activation of endothelial tip cells that migrate towards VEGF signals and is supported by stalk cell proliferation. Delta-like ligand, DLL4, is expressed on the tip cell which binds to Notch receptors on the stalk cells preventing their transformation into tip cells^6^. Notch signaling shuts down adjacent cells to the tip cell causing adequate spacing between sprouts, whereas blockage of Notch signaling results in a dramatic increase of sprouts, branching, and filopodia extension^8^. Stalk cells proliferate to extend the sprout towards the VEGF gradient and eventually these tip cells reestablish connections with previously established vasculature to form a closed network.

The initiation of sprouting angiogenesis and the onset of blood flow through the neovasculature leads to increased tissue oxygenation, tumor survival, and cancer progression. Many drugs have shown promise for their use in anti-angiogenic therapy, especially when combined with other drugs, typically cytotoxic chemotherapy. However, there are still considerable difficulties that need to be overcome, such as drug resistance, promotion of metastasis, and toxicity^9^. Computational modeling and multiscale systems biology can be effective tools for modeling sprouting angiogenesis and for the prediction of potent anti-angiogenic treatments for reducing tumor size, inhibiting or slowing growth. Modeling can help elucidate the contributions of endothelial cell proliferation and migration to vascular coverage, thus enabling the prediction of which mechanism would be the most effective for drug targeting.

There are several *in vitro* ways to access proliferation and migration of endothelial cells. A common way of measuring proliferation is with BrdU, which living cells incorporate into their DNA and allows them to be counted^10^. Colorimetric proliferation assays are also common, such as WST-1, cell counting kit-8 assay, which causes the reduction of formazan dye in proportion to the number of living cells and can be measured with a fluorescence plate reader^11^,^12^. Migration can be measured in real time using an RTCA reader based on electrical impedance^13^. Wound healing type assays are also used, where cells are plated with a stopper in the center of the chamber, which is then removed. After a certain number of hours, the cells that have migrated within that region can be counted^11^,^12^.

A significant class of studies have been performed in the area of computational modeling of angiogenesis (for reviews see^14^,^15^) and as it relates specifically to tumor growth (for reviews see^16^,^17^,^18^). The migration of tip cells, both chemotactic and haptotactic, was examined in response to differences in angiogenic factor distributions^19^. A cellular Potts model was used to understand the growth of vasculature under different extracellular matrix (ECM) conditions^20^. A significant number of studies have focused on the tumor vasculature in regards to drug delivery for cancer treatment^21^. Computational models have examined the effects of interstitial and vessel pressure on drug delivery^22^, transport of different drugs on tumor treatment^23^, delivery of anti-angiogenic drugs on tumor growth^24^, as well as examining the role of vasculature and drug delivery on drug resistance^25^. These models help us understand the overall process of angiogenesis but in this study we are interested in the specific contributions of endothelial cell migration and proliferation.

The importance of endothelial cell migration and proliferation has been a topic of several modeling studies. Burke *et al*. used a 2D lattice-based model to study endothelial cell proliferation and migration under mechanical stress^26^. They determined that the changes seen under mechanical stress could only be reproduced by increasing migration, increasing proliferation and having biased endothelial cell migration perpendicular to the direction of the strain. Another model used partial differential equations to study angiogenesis as an interplay between cell adhesion, traction force (elongation), and proliferation; the authors found that straight (non-tortuous) vasculature was found when proliferation was triggered by endothelial cell strain^27^. This model focuses on mechanical strain under *in vitro* conditions, rather than in a tumor microenvironment. In a cellular Potts model, migration and growth were considered to be modulated by blood flow, without which the vasculature would collapse^28^; the model did not examine the individual contributions of endothelial cell elongation, proliferation and migration.

Popel and his colleagues in a series of studies^29^,^30^ have formulated a class of 3D models of angiogenesis at multiple scales. In parallel with these 3D simulations, whole-body compartmental models have been used to predict VEGF distributions and VEGF receptor occupancy^31^,^32^ as well as pharmacokinetics and pharmacodynamics of anti-angiogenic drugs, such as bevacizumab and aflibercept^17^,^31^,^33^,^34^. Other models focused on the effects of vascular endothelial growth factor (VEGF) and Delta-like 4 Notch ligand on angiogenic tip sprouting^29^,^35^. In this paper, we develop a modified version of the angiogenic tip sprouting module, including tip cell migration, endothelial cell proliferation, and sprouting. This model is then used to understand the interplay between endothelial cell proliferation and migration rates in tumor angiogenesis. Specifically, the model investigates the therapeutic effects of increasing or decreasing endothelial cell migration and proliferation. Thus, we can use this model to predict angiogenic response to therapeutics affecting migration or proliferation. The problem of the relative contributions of proliferation and migration is very important for the general understanding of the regulation of angiogenesis, and also for translational applications since there are pharmacological agents that can differentially affect these phenomena. For example, there are anti-angiogenic peptide agents that preferentially affect blood or lymphatic endothelial cell proliferation and migration^36^,^37^. It should also be noted that these results would be important in the field of therapeutic angiogenesis where the growth of blood vessels is stimulated to provide blood flow and oxygen delivery to ischemic tissues, e.g. in the case of coronary and peripheral artery diseases, wound healing, and regenerative medicine^38^. Thus, the results of this study could also provide guidance to pro-angiogenic therapeutic treatments.

## Results

Simulations were performed on a 500 × 500 × 500 micron grid with the initial capillaries located along the edges of the cube. The proliferation and migration rates were varied for each simulation and the different simulation runs were averaged over 10 different trials. We will use the following metrics to characterize the vasculature similar to those used in describing vascular networks in tumor xenografts^39^. These are: Vascular Length Density, VLD, in mm/mm^3^; Bifurcation Density, BD, in 1/mm^3^; Vascular Segment Length, VSL, (length of vascular segment between adjacent bifurcations) in mm; Vascular Segment Tortuosity, VST, (total distance between bifurcations/Euclidean distance between bifurcations) in mm/mm; Fractal Dimension (FD). We will report mean values and variation of these variables and also where appropriate their distributions. Note that in characterizing vascular tortuosity we follow the morphological definition in ref. 40 rather than an alternative functional definition in ref. 39.

**Figure 1:**
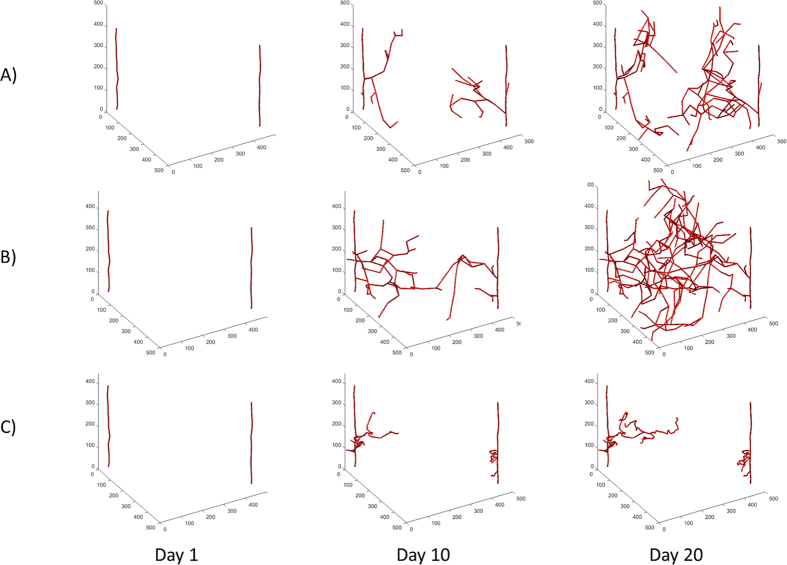
Simulations of Vascular Growth Over Time. (**A**) Example simulation with low proliferation, PR = 0.015. The vessels do not fill the space and are less tortuous. (**B**) Example simulation with medium proliferation rate, PR = 0.025 1/hr. The vessels are less tortuous and fill the space. (**C**) Example with high proliferation, PR = 0.083. The vessels do not fill the space and are very tortuous. In all cases the migration rate is MR = 10 μ/hr.

Sprouting Angiogenesis Progression over Time: We performed simulations under different parameter values and calculated the progression of the emerging vasculature over time. The motivation and focus of this investigation is on how the proliferation (PR) and migration rates (MR) affect vasculature, since these rates can be differentially attenuated by selective agents in proliferative diseases like cancer; alternatively the rates can be differentially increased by agents in the case of ischemic diseases, wound healing or regenerative medicine. In these simulations, VEGF is at a constant value of 20 ng/ml. Figure 1 shows the progression at days 1, 10 and 20 with a fixed migration rate, MR = 10 μ/hr and three different values of the proliferation rate, PR = 0.0147 (A), 0.025 (B), 0.083 (C) 1/hr (corresponding to divisions every 68, 40, and 12 hours, respectively). Doubling times for microvascular endothelial cells (MEC) range from about 12 hours to 4 days^41^,^42^,^43^ and human umbilical vein endothelial cells (HUVEC) range from 17 to 72 hours^44^,^45^,^46^. PR has a major effect on the progression of the tumor vasculature. At day 20, the values of parameters introduced above are: VLD = 72.4, 130.6, 16.9 mm/mm^3^, VSL = 0.193, 0.233, 0.124 mm, and VST = 1.37, 1.41, 2.60 mm/mm, respectively. When PR is low, the vessels reach the interior of the tumor space but the capillaries do not cover much of the area, Fig. 1A; in this case the VLD, VSL, and VST values are intermediate. Thus, migration allows the vasculature to extend into the tumor space but with fewer cells and thus fewer bifurcations covering the tumor space. When PR is medium, the tumor vasculature spreads throughout the entire region and has high VLD and VSL values and an intermediate VST, Fig. 1B. In this case, there is a balance between proliferation and migration, where proliferation is small enough to allow for the extension of the tip cell agent but fast enough for the vessels to grow as well. This leads to extensions into the tumor space due to migration and better coverage due to branching off of the new cell agents. When PR is high, the resulting vasculature is minimal but very tortuous with a low VLD and VSL and high VST level. The vasculature does not fully extend into the interior of the tumor space, Fig. 1C. This is partially due to the fact that the high PR limits the extension of the vasculature and so the capillaries do not spread into the interior. The growth is further limited by anastomosis; since the sprouts are short they end up growing into another tip cell agent and anastomosing, which in turn limits sprouting due to vessel regression. In general, the vasculature is the fullest at medium values of PR and is most tortuous at high values of PR. We have also performed simulations in the presence of a VEGF gradient to explore the effect of the gradient, see Supplemental Data for more information.

**Figure 2:**
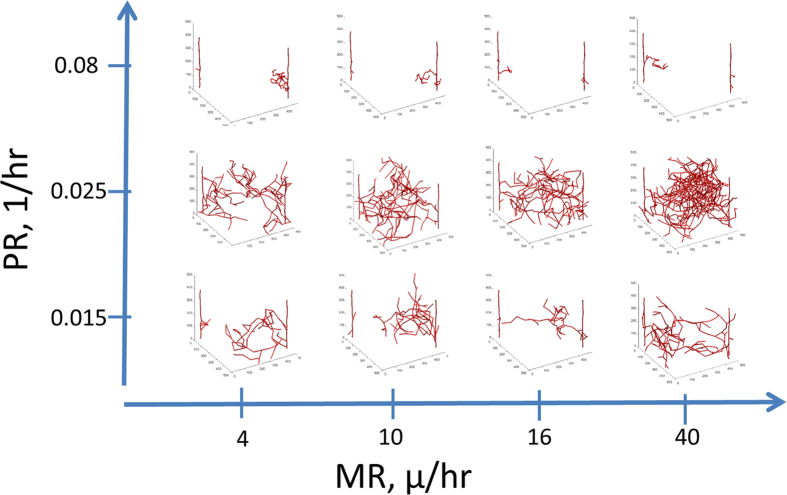
Effects of Proliferation Rate (PR) and Migration Rate (MR) on the vasculature at day 20. With low MR, there is less growth of vasculature. When MR is high and PR is low, there is more vascular coverage but it does not fill the space. The optimal coverage occurs at medium amounts of proliferation and migration. When PR is high, the vessels are very tortuous and do not cover the space.

Medium Proliferation Values Produce the Most Vascular Coverage: In Fig. 2 we show simulation images with different PR and MR at day 20. Day 20 is chosen here since it is relevant to the timeframe of tumor xenograft growth that will be used in comparison with experimental data below. With low PR and MR values, the vasculature is very minimal. Having high PR also results in very minimal architectures that are very tortuous. With low PR and high MR, the capillaries are long but there are not many sprouts and the tumor space is not well covered. With medium amounts of PR and MR, there is complete coverage of the tumor space. Here it is clear that the vasculature reaches the middle of the space and the density of the vasculature is not too high. Thus, these results suggest that medium proliferation values would be optimal for cancer vascular recruitment.

**Figure 3:**
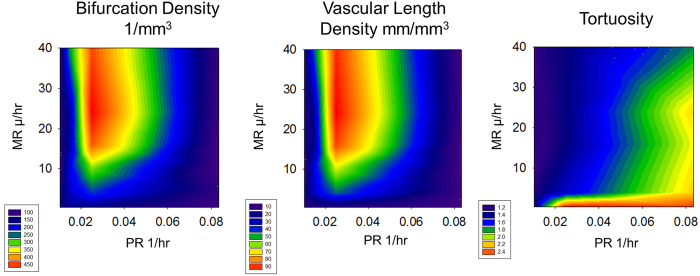
Contour Plots of Vascular Metrics at 20 Days. We show a contour plot of the number of bifurcations per mm^3^, the vascular length density in mm/mm^3^ and tortuosity for different MR and PR. The number of bifurcations and the length density depend mostly on proliferation whereas tortuosity depends on both parameters.

Most Vascular Metrics are Determined by Proliferation: To better understand the contribution and synergy of PR and MR in the sprouting angiogenesis model, we conducted a parameter space evaluation, varying MR between 0.24 and 40 μ/hr and PR between 0.015 and 0.083 1/hr for 20 days, Fig. 3. We find that the bifurcation density (BD) and the vascular length density (VLD) are mostly dependent on proliferation. There is an optimal PR value of about 0.025 where the BD and VLD are highest except at very low MR. At very low or very high PR, the BD is low. At low PR, there are fewer cells and therefore there are fewer places to sprout, resulting in a reduction of the number of bifurcations. At high PR, there tend to be too many sprouts formed over a short amount of time resulting in anastomosis, reducing the number of bifurcations and the number of vascular segments. Larger or smaller PR values cause a decrease in BD and VLD. In terms of treatment strategy, this would mean that decreasing proliferation would be an effective strategy for limiting vasculature. Surprisingly though, increasing proliferation would also be an effective strategy. This is due to the fact that highly proliferative vessels tend to anastomose quickly limiting their overall growth. Tortuosity (VST) is dependent on both PR and MR. The vasculature is most tortuous at very low MR or very high PR. This is due to the fact that these values lead to the shorter segments that curve due to their random walk.

Comparison with Experimental Data: We compare our model results to *in vivo* data derived from a breast cancer xenograft model, where the mouse vasculature was perfused with a polymer solution and excised tumors were imaged with microCT for the purpose of visualizing the entire tumor vascular bed^39^,^47^. A digital map of the vascular network was obtained with a specially developed 3D reconstruction algorithm. Thus, 3D reconstructions of the entire xenograft tumors were able to be developed and vascular metrics were calculated for these tumors (Imaging Data). These metrics were also compared to data from the literature (Literature Values). Several metrics were used to characterize the vasculature, such as vascular length density, vascular segment length, bifurcation density, tortuosity, and fractal dimension, Table 1. We also present the ranges of these parameters for different tumors from the literature. The simulation results generally fall within the range of those seen *in vivo*. More detailed and focused simulations are necessary to simulate a specific tumor type.

Regression Distance does not Affect Mean Vessel Length: We varied the distance *d*_*r*,_ in which vessels growing near a mature vessel would regress, from 50 to 200 microns. Any new sprout within this distance of a mature vessel would retract, the base example being 100 microns. Using a radial distance of 200 microns, we found overall that the mean BD and VLD were lower than normal, 136 vs 194 bifurcations per mm^3^, and 18 vs 27.9 mm/mm^3^. In contrast, the VSLs were comparable: 0.107 and 0.104 mm. There were opposite effects using a regression distance of 50 microns. BD increased from 194 to 315 and VLD increased from 27.9 to 43.9 mm/mm^3^. Once again the VSL were the same 0.104 mm.

**Figure 4:**
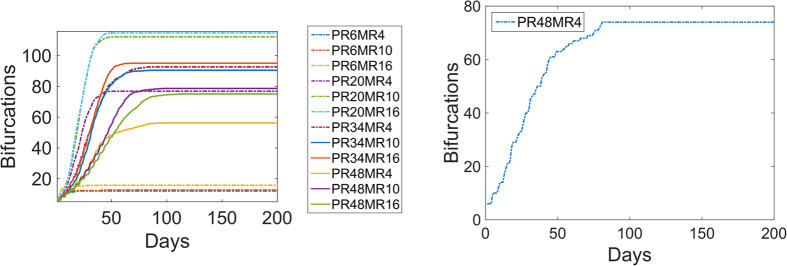
Mean Bifurcation Density Over Time. (**A**) We performed simulations until they reach steady state and show the mean bifurcation density over time for different PR and MR. There is a set of high proliferation values (PR = 0.083) that results in a low number of bifurcations. (**B**) An example simulation showing the numbers of bifurcations reaching a plateau at around 80 days. PR = 0.010 1/hr and MR = 4 μ/hr.

Vascular Network Characteristics as Functions of Time: In the previous simulations we presented the results for t = 20 days; this time point was chosen as a typical duration for tumor xenografts, eg^39^. However, it is of interest to investigate how the network characteristics vary with time as the vasculature continues to evolve. Thus, we ran the simulations for 200 days. We plotted the results for BD averaged over 10 runs as functions of time and found that all simulations reached a steady state, Fig. 4A; we also plotted a sample simulation, Fig. 4B, that exhibits fluctuations associated with sprout formation and retraction. Most of the simulation values resulted in high BD (200 or above) but there were a small sample of cases where there were very few bifurcations <20. The cases with low BD all had high PR values of 0.083, corresponding to proliferating every 6 hours. In conclusion, the limited number of bifurcations at high PR values does not change with more time.

**Figure 5:**
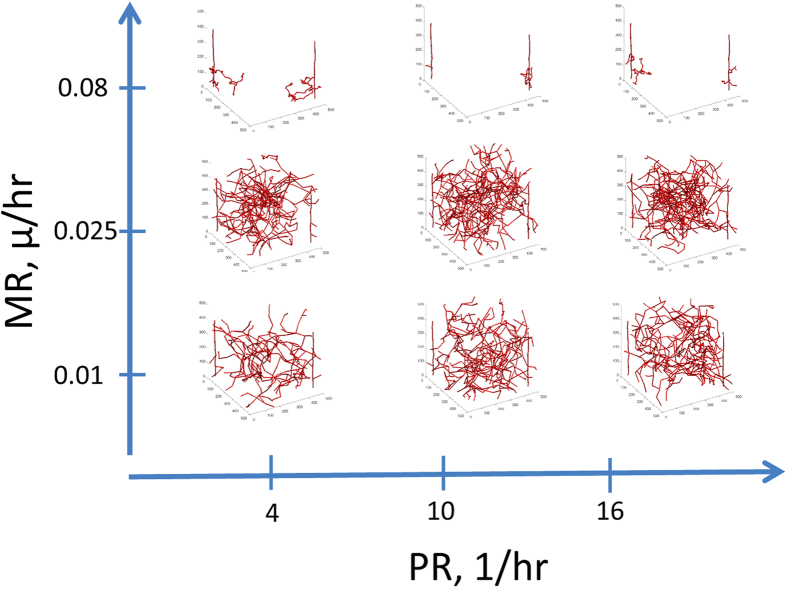
Simulations for Different Proliferation and Migration Rates at Steady State. We show examples of the steady state images at 50 days. At very high PR the vasculature is stunted and does not fill the space. At all other values, the vasculature fills the space.

To further elucidate the changes with respect to time, in Fig. 5 we show simulation images under different PR and MR conditions at 50 days, similar to the same variables at 20 days in Fig. 2. As described before, under most conditions the vasculature completely covers the tumor space. Only at very high levels of proliferation does the vasculature become shunted and non-functional. This supports the notion that surprisingly increasing endothelial cell proliferation might actually decrease vascular coverage, although this effectiveness might be limited to small tumors that have recruited very little vasculature.

## Discussion

Computational models can be useful in formulating hypotheses, especially when care is taken to justify each abstraction made and to reconcile it with biological reality. This task is made easier by the discrete modeling of endothelial cells as agents capable of independent decisions as well as inter-agent actions. Discrete models like this can serve as a framework for the type of module-based modeling previously described^14^,^29^,^30^ which attempt to combine independently validated models of cellular processes as modules in a larger integrated model of angiogenesis, or can be incorporated into other models as processes such as tumor growth or metastatic colonization in which angiogenesis is involved.

It is well known that tumor vasculature is abnormal and differs from the normal vasculature of the host organ^48^,^49^. One of the abnormalities is that the vessels are very tortuous^50^ in and around the tumor. Endothelial cell proliferation and migration are controlled by a myriad of factors in the tumor microenvironment including a large number of pro- and anti-angiogenic factors that could be used for pro- and anti-angiogenic therapy^51^. Therefore in an experimental setting it is very difficult to modulate or separate out these two processes. In contrast, it is possible to modulate proliferation and migration rates separately in a computational model. Thus, we examined the effects of both of these properties on the characteristics of a growing vascular network.

The overall growth of vasculature is a result of both proliferation and migration. Cell migration in the model is a combination of elongation and motility, such that it includes elongation because the length of the tip cell agent gets longer. It also includes motility such that the leading node of the tip agent can change direction and the rate in which it migrates. It should be noted that the motility of a tip cell does not directly relate to isolated cell migration measured *in vitro*, such that isolated cells moving on a plate or in 3D matrix may not follow the same dynamics as a tip cell migrating with stalk and phalanx cells behind it. In the model we show that when either proliferation or migration is acting on its own, the vasculature is shunted and ineffective at covering the tumor space. This can be seen by comparing the VLD, at low PR with high MR yields VLD of around 20–30 mm/mm^3^, high PR with low MR rate yields VLD of around 10 mm/mm^3^. whereas an intermediate value of both yields a VLD of around 80–90 mm/mm^3^. Thus, neither proliferation nor migration alone can create organized vascular growth; a balance is necessary between the two processes.

Comparing the contour plots of the parameter space with the representation of the tumor vasculature, we find interesting results. When examining the contour plot for VLD, it is clear that values with high PR and low to medium MR result in large values of total vasculature. When examining the parameter space, simulations with low PR do not have large BD or VLD. High levels of PR actually decrease the BD and VLD. This suggests that tumors that give uncontrolled proliferatory signals might actually cause the vasculature to be sparse in certain areas of the tumor resulting in areas of hypoxia. Thus, controlling proliferation through vascular normalization^52^, may improve the vessel coverage and improve dysfunctional vasculature and drug delivery.

We can relate our results to work done by Lee *et al*. who analyzed the effects of anti-angiogenic peptides on the proliferation and migration of endothelial cells^11^. For example, somatotropin-derived peptide SP5028 inhibits migration of microvascular endothelial cells (at 50 μM concentration at 20 h) by 70% and is also a potent proliferation inhibitor; thus, our model predicts that it would be an effective angiogenic inhibitor. Also, another peptide from the same class, SP5031, is a less potent proliferation inhibitor, but it reduces migration by >90% which is predicted to be effective at inhibiting angiogenesis. In agreement with these data, the peptide SP5031 significantly reduced VEGF-induced angiogenesis, which was measured by staining with CD34 in an *in vivo* subcutaneous Matrigel plug assay. Using a collagen-derived biomimetic peptide SP2043, a 95% reduction in migration would inhibit bifurcations and vascular segment density enough to inhibit angiogenesis so treating with 25 μM of SP2043 is predicted to be sufficient to inhibit angiogenesis in agreement with experimental data^53^.

The major prediction of our model is that PR would have a greater effect than MR on reducing vascular growth. In support of this conclusion, Jackson and colleagues found that proliferation is necessary for the extension of the vasculature otherwise the capillary ceases to extend^54^. Very low MR causes a reduction in BD and SLD but overall proliferation has more of an effect on these metrics. Tortuosity is governed by both PR and MR, such that high tortuosity occurs at very high PR or very low MR. This makes sense because migration causes vessels to be straighter^7^ and thus low migration reduces this; high proliferation limits BD because it limits the extension of each tip cell agent. Interestingly, our model predicts that increasing PR might also be an effective strategy at reducing vasculature, as it leads to an increase in sprouts which then anastomose to create non-functional vessels. Others have found that increasing branching can lead to tumor growth inhibition by forming non-productive angiogenesis^55^,^56^. This is in line with the commonly seen effect of tumor vasculature being tortuous and non-functional, possibly due to over-proliferation.

There are several limitations of the current model. For one, we consider a tumor space that is constant and unchanging, whereas in reality the tumor would grow over time with the recruitment of new vasculature. Thus, we are focused on a section of a tumor that is recruiting new vasculature but not focusing on the expansion of the tumor. The interplay between the tumor and vasculature should be the subject of further studies. Another limitation of this model is that we have explicitly included the extracellular matrix and interplay with matrix metalloproteinases (MMPs). This is an important process but beyond the scope of this study. Another limitation is that we only examine VEGF-A with the assumption that it consists of isoform VEGF_164_. In the present analysis we do not distinguish between different VEGF isoforms, e.g. VEGF_121_, VEGF_165_ and VEGF_189_. However, it is well established that the patterns of vascular network formation depends on the differential isoform expression^7^, the current model will need to be expanded to incorporate these differences.

In conclusion, this model examines the complex interplay between tip cell migration and stalk cell proliferation. Specifically, we demonstrate that proliferation greatly influences total vascular coverage and, thus, may be a more effective target for anti-angiogenic therapies. These findings highlight the utility of computational modeling in parsing the critical components of complex biological processes, such as angiogenesis, to better optimize therapeutic development and the identification of potentially synergistic drug combinations.

## Methods

### Model Overview

The capillary sprouting model presented here is a hybrid agent-based model that models endothelial cells (EC) as physical agents on a discrete three-dimensional grid. The coordinates of the agents and events such as proliferation and migration occur in continuous space but are mapped to a discrete grid for representation and storage of environmental values. The model takes advantage of the well-developed agent-based and probabilistic approaches to modeling directional angiogenesis in order to produce realistic capillary morphologies under various settings. The general flow of the code is illustrated in Supplementary Figure 1: tip cell migration and anastomosis, stalk cell proliferation and anastomosis, sprouting and vessel regression.

### Endothelial Cell Agent

The environment of the *in silico* model is a discrete 500 × 500 × 500 micron grid. Each grid coordinate contains environmental variable [VEGF] and may be occupied by an agent. The default VEGF concentration is assumed to be a constant value of 20 ng/ml. The environmental grid represents the ‘tumor space.’ We have also examined several different VEGF gradients, see Supplementary Figure 1. Endothelial cells (EC) are the main components of blood vessels and each agent in the model is assigned an endothelial cell type, either tip, stalk, or phalanx, and comprises a forward node (Node 2) and trailing node (Node 1). An agent ahead of another agent would share its trailing node coordinate with the forward node of the agent behind it, thus forming continuous capillaries. This agent definition is an extension of^35^; along with node coordinates, agents also store values for radius, activation level, phenotype, and cell cycle length. Each endothelial cell is made up of a group of agents. Sprouting can only occur once for each endothelial cell.

### Initial Capillaries

The initial configuration consists of two capillaries at diagonal ends of the tumor space. These capillaries are mature and do not proliferate or migrate. Each capillary is made up of ten endothelial cell agents. During the first iteration, an endothelial agent in each of the capillaries forms a sprout, creating a new tip cell. This is the start of sprouting angiogenesis in the simulation. The simulation runs for twenty days (or 50 days for comparison), similar to the length of time of an *in vivo* xenograft model.

### Tip Cell Decisions

#### Proliferation

A tip cell agent only proliferates if it has sprouted, creating a bifurcation, and thus needs to form a stalk cell agent. It must wait until it has reached its cell cycle to proliferate. The amount of time it takes to complete a cell cycle is chosen from a normal distribution with a mean P_s_ and standard deviation σ_s_. These values are presented in Supplementary Table 1. The former tip cell agent becomes the new stalk cell agent and a new agent is created which becomes the new tip cell agent. The new tip cell extends in the direction of the old tip agent. Once the tip cell agent has proliferated to form the stalk cell agent, it no longer proliferates.

#### Migration

Migration is the primary means by which the growing sprout is able to change directions in response to chemotactic and haptotactic cues. Migration is limited to tip cell agents in this model. Many previous models have modeled angiogenesis as a reinforced random walk or a diffusion process^57^. We model migration the same way as was done previously^35^:


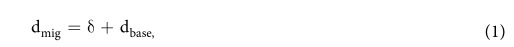


where d_base_ is the base endothelial cell migration rate in μ/hr. Thus the tip cell agent migrates by d_mig_ in the direction chosen by the filopodia search. Migration is limited, such that an agent can only extend 50% of its current length. The maximum total length of an agent is limited to d_max_. The values of parameters are presented in Supplementary Table 1.

The search for the direction of tip cell migration includes persistence. Persistence is defined as


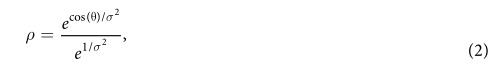


where *θ* is the angle between the current tip cell agent’s direction and the direction being considered, and σ is π/6^57^. The direction score of each direction during the search of the local environment is 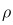.


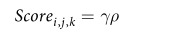


where γ is the weight of persistence.

Thus


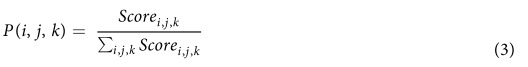


is the probability of moving in the direction (i,j,k).

In this model, tip cell agents migrate each iteration, attempting to reorient themselves according to equation (3)^58^. It is likely the case that the tip cell blazes a path forward by releasing matrix metalloproteinases (MMP) that degrade the surrounding ECM and by secreting components of basement membrane such as collagens^59^.

#### Stalk Cell Proliferation

Proliferation is based on the individual cell cycle that must be completed before proliferating. The amount of time it takes to complete a cell cycle is chosen as above. These values are varied based on the cell’s proliferation rate.

Proliferation is implemented first by creating a new agent. The forward node of the new tip cell agent is positioned in the same direction as the old tip in the normalized direction of the old tip cell agent. The tip cell agent remains active and the stalk cell agent deactivates. The new agent is assigned a cell cycle duration. Each new agent is part of a larger endothelial cell unit, thus once the total length of the endothelial cell unit is greater than e_min_ the new stalk agent become part of a new endothelial cell unit. The stalk cell agent only proliferates if the new tip agent does not leave the gridspace. After the stalk cell agent proliferates, it determines whether it will anastomose (see section on anastomosis).

#### Sprouting

Sprouting is determined probabilistically as long as the new sprout will not be on the boundary. A stalk cell agent must be at the beginning of an endothelial cell unit to bifurcate and form a sprout. The trailing node of the new sprout is the position of the forward node of the sprouting capillary agent and the direction of the forward node of the new sprout is determined randomly. This new agent is an activated tip cell agent and the first cell in a new capillary. The tip cell agent then proliferates to form a stalk cell. Once sprouting has occurred the cells attached to the sprouting capillary cell deactivate and are no longer able to sprout. This is due to Delta-Notch signaling in sprouting angiogenesis which has been demonstrated *in vivo* and *in silico*^60^.

#### Anastomosis

Anastomosis can form in one of two ways: 1) when two tip cell agents migrate into one another they can anastomose and form a connection between the two capillaries; and 2) when a tip cell agent migrates into a non-tip cell (either a stalk agent or a phalanx cell) and they fuse. The two different processes are essentially the same with a few minor modifications. Specifically when a migrating tip cell agent approaches another tip cell, the migrating tip agent’s forward node is assigned to the location of the forward node of the position of the other tip cell agent, essentially connecting the two tip cells at their ends. This is logical because the filopodia of two tip cells will sense one another and reorient the migrating tip cell to attach to its end. When a migrating tip cell hits any other cell (either a stalk cell agent or a quiescent phalanx cell), the migrating tip cell agent’s forward node will be placed at the point in the grid where they hit and the two capillaries will fuse at this point. The tip cells deactivate when they anastomose. Both capillaries that have anastomosed become mature, such that the cells in each capillary are no longer allowed to proliferate or migrate.

#### Sprout Regression

Endothelial cells can sense the level of VEGF; if a sprout is close to a functional vessel (with blood flow under *in vivo* conditions), oxygen is delivered locally by the functional vessel that would prevent VEGF elevation in the vicinity of the sprout via HIF1α mechanism. We include sprout regression if a newly formed sprout comes close to a functional vessel. Specifically, we assume that if there is a functional vessel within a distance *d*_*r*_ from the tip, other than the capillary from which it originated, the sprout will no longer grow and will regress; we will refer to *d*_*r*_as regression distance. The values of d_*r*_are varied within a range shown in Supplementary Table 1 to study the sensitivity of the results to this parameter. Two mature capillaries within a distance *d*_*r*_ from each other would have overlapping areas of diffusion. So newly formed sprouts within the regression distance *d*_*r*_ from another functional capillary would feel a smaller oxygen gradient, causing regression. On the other hand, sprouts that are farther than the distance *d*_*r*_ from a mature vessel would sense a stronger gradient and would continue to grow.

## Additional Information

**How to cite this article**: Norton, K.-A. and Popel, A. S. Effects of endothelial cell proliferation and migration rates in a computational model of sprouting angiogenesis. *Sci. Rep*. **6**, 36992; doi: 10.1038/srep36992 (2016).

## Supplementary Material

Supplementary Information

## Figures and Tables

**Table 1:** A Comparison of Vascular Metrics.

Metric	Units	Imaging Data, cited in [47]	Model Values, 20 Days	Model Values, Steady State	Literature Values, cited in [47]
Vascular length density*	mm/mm^3^	8.73–101.18	2.5–95.4 (27.9)	7.66–234 (130)	10–72
Tortuosity*	mm/mm		1.10–2.52 (1.58)	1.29–2.92 (1.616)	
Vascular segment length*	mm	0.13–0.43	0.04–0.17 (0.104)	0.07–0.23 (0.17)	0.06–0.3
Bifurcation density*	1/mm^3^	40.1–231.5	60–493 (194)	97–918 (555)	NA
Fractal dimension*	—	1.01–2	1.57–1.91 (1.72)		1.94–2.04

We compare a range of vascular metrics from our simulation to 3D vascular reconstruction data from whole tumor xenografts (Imaging Data). Our values are comparable with those in the tumor xenografts and other literature.

## References

[b1] Hanahan D. & Weinberg R. A. Hallmarks of cancer: the next generation. Cell 144, 646–674 (2011). 21376230 10.1016/j.cell.2011.02.013

[b2] Hanahan D. & Weinberg R. A. The hallmarks of cancer. Cell 100, 57–70 (2000). 10647931 10.1016/s0092-8674(00)81683-9

[b3] Folkman J. Angiogenesis: an organizing principle for drug discovery? Nature reviews. Drug discovery 6, 273–286 (2007). 17396134 10.1038/nrd2115

[b4] Chappell J. C., Wiley D. M. & Bautch V. L. Regulation of blood vessel sprouting. Seminars in cell & developmental biology 22, 1005–1011 (2011). 22020130 10.1016/j.semcdb.2011.10.006 PMC4521217

[b5] Claesson-Welsh L. & Welsh M. VEGFA and tumour angiogenesis. Journal of internal medicine 273, 114–127 (2013). 23216836 10.1111/joim.12019

[b6] Chung A. S., Lee J. & Ferrara N. Targeting the tumour vasculature: insights from physiological angiogenesis. Nature reviews. Cancer 10, 505–514 (2010). 10.1038/nrc2868 20574450

[b7] Vempati P., Popel A. S. & Mac Gabhann F. Extracellular regulation of VEGF: isoforms, proteolysis, and vascular patterning. Cytokine & growth factor reviews 25, 1–19 (2014). 24332926 10.1016/j.cytogfr.2013.11.002 PMC3977708

[b8] Blanco R. & Gerhardt H. VEGF and Notch in tip and stalk cell selection. Cold Spring Harbor perspectives in medicine 3, a006569 (2013). 23085847 10.1101/cshperspect.a006569 PMC3530037

[b9] Vasudev N. S. & Reynolds A. R. Anti-angiogenic therapy for cancer: current progress, unresolved questions and future directions. Angiogenesis 17, 471–494 (2014). 24482243 10.1007/s10456-014-9420-y PMC4061466

[b10] Noren D. P. . Endothelial cells decode VEGF-mediated Ca2+ signaling patterns to produce distinct functional responses. Science signaling 9, ra20 (2016). 10.1126/scisignal.aad3188 PMC5301990 26905425

[b11] Lee E., Rosca E. V., Pandey N. B. & Popel A. S. Small peptides derived from somatotropin domain-containing proteins inhibit blood and lymphatic endothelial cell proliferation, migration, adhesion and tube formation. The international journal of biochemistry & cell biology 43, 1812–1821 (2011). 21920451 10.1016/j.biocel.2011.08.020 PMC3206162

[b12] Rosca E. V. . A biomimetic collagen derived peptide exhibits anti-angiogenic activity in triple negative breast cancer. PloS one 9, e111901 (2014). 25384034 10.1371/journal.pone.0111901 PMC4226498

[b13] Norton K. A., Popel A. S. & Pandey N. B. Heterogeneity of chemokine cell-surface receptor expression in triple-negative breast cancer. American journal of cancer research 5, 1295–1307 (2015). 26101698 PMC4473311

[b14] Noren D., Rekhi R., Long B. & Qutub A. A. In Vascularization: Regenerative Medicine and Tissue Engineering. (ed. Brey E. M.) 213 (2014).

[b15] Peirce S. M., Mac Gabhann F. & Bautch V. L. Integration of experimental and computational approaches to sprouting angiogenesis. Current opinion in hematology 19, 184–191 (2012). 22406822 10.1097/MOH.0b013e3283523ea6 PMC4132663

[b16] Enderling H. & Rejniak K. A. Simulating cancer: computational models in oncology. Frontiers in oncology 3 (2013). 10.3389/fonc.2013.00233 PMC3772565 24062986

[b17] Finley S. D., Chu L.-H. & Popel A. S. Computational systems biology approaches to anti-angiogenic cancer therapeutics. Drug discovery today 20, 187–197 (2015). 25286370 10.1016/j.drudis.2014.09.026 PMC4336587

[b18] Stefanini M. O., Qutub A. A., Mac Gabhann F. & Popel A. S. Computational models of VEGF-associated angiogenic processes in cancer. Mathematical Medicine and Biology dqq025 (2011). 10.1093/imammb/dqq025 PMC4104688 21266494

[b19] Vilanova G., Colominas I. & Gomez H. Capillary networks in tumor angiogenesis: from discrete endothelial cells to phase-field averaged descriptions via isogeometric analysis. International journal for numerical methods in biomedical engineering 29, 1015–1037 (2013). 23653256 10.1002/cnm.2552

[b20] Daub J. T. & Merks R. M. A cell-based model of extracellular-matrix-guided endothelial cell migration during angiogenesis. Bulletin of mathematical biology 75, 1377–1399 (2013). 23494144 10.1007/s11538-013-9826-5 PMC3738846

[b21] Kim M., Gillies R. J. & Rejniak K. A. Current advances in mathematical modeling of anti-cancer drug penetration into tumor tissues. Front Oncol 3, 278 (2013). 24303366 10.3389/fonc.2013.00278 PMC3831268

[b22] Tang L. . Computational modeling of 3D tumor growth and angiogenesis for chemotherapy evaluation. PloS one 9, e83962 (2014). 24404145 10.1371/journal.pone.0083962 PMC3880288

[b23] Magdoom K. N. . MRI-based computational model of heterogeneous tracer transport following local infusion into a mouse hind limb tumor. PloS one 9, e89594 (2014). 24619021 10.1371/journal.pone.0089594 PMC3949671

[b24] Gevertz J. Optimization of vascular-targeting drugs in a computational model of tumor growth. Physical review. E, Statistical, nonlinear, and soft matter physics 85, 041914 (2012). 10.1103/PhysRevE.85.041914 22680505

[b25] Spill F., Guerrero P., Alarcon T., Maini P. K. & Byrne H. M. Mesoscopic and continuum modelling of angiogenesis. Journal of mathematical biology 70, 485–532 (2014). 24615007 10.1007/s00285-014-0771-1 PMC5320864

[b26] Burke D. & Kelly D. J. A mechanobiological model of endothelial cell migration and proliferation. Computer methods in biomechanics and biomedical engineering 19, 74–83 (2016). 25513718 10.1080/10255842.2014.989388

[b27] Santos-Oliveira P. . The Force at the Tip–Modelling Tension and Proliferation in Sprouting Angiogenesis. PLoS Comput Biol 11, e1004436 (2015). 26248210 10.1371/journal.pcbi.1004436 PMC4527825

[b28] Bazmara H. . The Vital Role of Blood Flow-Induced Proliferation and Migration in Capillary Network Formation in a Multiscale Model of Angiogenesis. PloS one 10, e0128878 (2015). 26047145 10.1371/journal.pone.0128878 PMC4457864

[b29] Liu G., Qutub A. A., Vempati P., Mac Gabhann F. & Popel A. S. Module-based multiscale simulation of angiogenesis in skeletal muscle. Theor Biol Med Model 8, 1–26 (2011). 21463529 10.1186/1742-4682-8-6 PMC3079676

[b30] Qutub A. A. & Popel A. S. Elongation, proliferation & migration differentiate endothelial cell phenotypes and determine capillary sprouting. BMC Syst Biol 3, 13 (2009). 19171061 10.1186/1752-0509-3-13 PMC2672076

[b31] Finley S. D. & Popel A. S. Effect of tumor microenvironment on tumor VEGF during anti-VEGF treatment: systems biology predictions. Journal of the National Cancer Institute 105, 802–811 (2013). 23670728 10.1093/jnci/djt093 PMC3672077

[b32] Tan W. H., Popel A. S. & Mac Gabhann F. Computational model of Gab1/2-dependent VEGFR2 pathway to Akt activation. PloS one 8, e67438 (2013). 23805312 10.1371/journal.pone.0067438 PMC3689841

[b33] Finley S. D., Angelikopoulos P., Koumoutsakos P. & Popel A. S. Pharmacokinetics of Anti-VEGF Agent Aflibercept in Cancer Predicted by Data-Driven, Molecular-Detailed Model. CPT: pharmacometrics & systems pharmacology 4, 641–649 (2015). 26783500 10.1002/psp4.12040 PMC4716581

[b34] Finley S. D. & Popel A. S. Predicting the effects of anti-angiogenic agents targeting specific VEGF isoforms. The AAPS journal 14, 500–509 (2012). 22547351 10.1208/s12248-012-9363-4 PMC3385824

[b35] Qutub A. A. & Popel A. S. Elongation, proliferation & migration differentiate endothelial cell phenotypes and determine capillary sprouting. BMC systems biology 3, 13 (2009). 19171061 10.1186/1752-0509-3-13 PMC2672076

[b36] Koskimaki J. E. . Serpin-derived peptides are antiangiogenic and suppress breast tumor xenograft growth. Transl Oncol 5, 92–97 (2012). 22496925 10.1593/tlo.11244 PMC3323930

[b37] Lee E., Koskimaki J. E., Pandey N. B. & Popel A. S. Inhibition of lymphangiogenesis and angiogenesis in breast tumor xenografts and lymph nodes by a peptide derived from transmembrane protein 45A. Neoplasia 15, 112–IN116 (2013). 23441126 10.1593/neo.121638 PMC3579314

[b38] Mac Gabhann F., Qutub A. A., Annex B. H. & Popel A. S. Systems biology of pro‐angiogenic therapies targeting the VEGF system. Wiley Interdisciplinary Reviews: Systems Biology and Medicine 2, 694–707 (2010). 20890966 10.1002/wsbm.92 PMC2990677

[b39] Stamatelos S. K., Kim E., Pathak A. P. & Popel A. S. A bioimage informatics based reconstruction of breast tumor microvasculature with computational blood flow predictions. Microvascular research 91, 8–21 (2014). 24342178 10.1016/j.mvr.2013.12.003 PMC3977934

[b40] Azegrouz H., Trucco E., Dhillon B., MacGillivray T. & MacCormick I. J. Thickness dependent tortuosity estimation for retinal blood vessels. Conference proceedings: … Annual International Conference of the IEEE Engineering in Medicine and Biology Society. IEEE Engineering in Medicine and Biology Society. Annual Conference 1, 4675–4678 (2006). 10.1109/IEMBS.2006.260558 17945850

[b41] Anagnostou A., Lee E. S., Kessimian N., Levinson R. & Steiner M. Erythropoietin has a mitogenic and positive chemotactic effect on endothelial cells. Proceedings of the National Academy of Sciences of the United States of America 87, 5978–5982 (1990). 2165612 10.1073/pnas.87.15.5978 PMC54453

[b42] Marin V., Kaplanski G., Gres S., Farnarier C. & Bongrand P. Endothelial cell culture: protocol to obtain and cultivate human umbilical endothelial cells. Journal of immunological methods 254, 183–190 (2001). 11406163 10.1016/s0022-1759(01)00408-2

[b43] Negre-Aminou P. . Inhibition of proliferation of human smooth muscle cells by various HMG-CoA reductase inhibitors; comparison with other human cell types. Biochimica et biophysica acta 1345, 259–268 (1997). 9150246 10.1016/s0005-2760(96)00184-1

[b44] Frye C. A. & Patrick C. W. Jr. Isolation and culture of rat microvascular endothelial cells. In vitro cellular & developmental biology. Animal 38, 208–212 (2002). 12197772 10.1290/1071-2690(2002)038<0208:IACORM>2.0.CO;2

[b45] Snead M. D., Papapetropoulos A., Carrier G. O. & Catravas J. D. Isolation and culture of endothelial cells from the mesenteric vascular bed. Methods in cell science 17, 257–262 (1995).

[b46] Zheng B. . Human myogenic endothelial cells exhibit chondrogenic and osteogenic potentials at the clonal level. Journal of orthopaedic research: official publication of the Orthopaedic Research Society 31, 1089–1095 (2013). 23553740 10.1002/jor.22335 PMC4360901

[b47] Kim E. . Multiscale imaging and computational modeling of blood flow in the tumor vasculature. Annals of biomedical engineering 40, 2425–2441 (2012). 22565817 10.1007/s10439-012-0585-5 PMC3809908

[b48] Carmeliet P. & Jain R. K. Principles and mechanisms of vessel normalization for cancer and other angiogenic diseases. Nature reviews. Drug discovery 10, 417–427 (2011). 21629292 10.1038/nrd3455

[b49] Goel S., Wong A. H. & Jain R. K. Vascular normalization as a therapeutic strategy for malignant and nonmalignant disease. Cold Spring Harbor perspectives in medicine 2, a006486 (2012). 22393532 10.1101/cshperspect.a006486 PMC3282493

[b50] Goel S. . Normalization of the vasculature for treatment of cancer and other diseases. Physiological reviews 91, 1071–1121 (2011). 21742796 10.1152/physrev.00038.2010 PMC3258432

[b51] Gacche R. N. & Meshram R. J. Angiogenic factors as potential drug target: efficacy and limitations of anti-angiogenic therapy. Biochimica et Biophysica Acta (BBA)-Reviews on Cancer 1846, 161–179 (2014). 24836679 10.1016/j.bbcan.2014.05.002

[b52] Huang Y., Stylianopoulos T., Duda D. G., Fukumura D. & Jain R. K. Benefits of vascular normalization are dose and time dependent–letter. Cancer research 73, 7144–7146 (2013). 24265277 10.1158/0008-5472.CAN-13-1989 PMC3876035

[b53] Lee E. . Inhibition of breast cancer growth and metastasis by a biomimetic peptide. Scientific reports 4, 7139 (2014). 25409905 10.1038/srep07139 PMC4238022

[b54] Jackson T. & Zheng X. A cell-based model of endothelial cell migration, proliferation and maturation during corneal angiogenesis. Bulletin of mathematical biology 72, 830–868 (2010). 20052558 10.1007/s11538-009-9471-1

[b55] Thurston G., Noguera-Troise I. & Yancopoulos G. D. The Delta paradox: DLL4 blockade leads to more tumour vessels but less tumour growth. Nature reviews. Cancer 7, 327–331 (2007). 17457300 10.1038/nrc2130

[b56] Miles K. M. . Dll4 blockade potentiates the anti-tumor effects of VEGF inhibition in renal cell carcinoma patient-derived xenografts. PloS one 9, e112371 (2014). 25393540 10.1371/journal.pone.0112371 PMC4231048

[b57] Plank M. J. & Sleeman B. D. A reinforced random walk model of tumour angiogenesis and anti-angiogenic strategies. Mathematical medicine and biology: a journal of the IMA 20, 135–181 (2003). 14636027 10.1093/imammb/20.2.135

[b58] Milde F., Bergdorf M. & Koumoutsakos P. A hybrid model for three-dimensional simulations of sprouting angiogenesis. Biophysical journal 95, 3146–3160 (2008). 18586846 10.1529/biophysj.107.124511 PMC2547461

[b59] Karagiannis E. D. & Popel A. S. Distinct modes of collagen type I proteolysis by matrix metalloproteinase (MMP) 2 and membrane type I MMP during the migration of a tip endothelial cell: insights from a computational model. Journal of theoretical biology 238, 124–145 (2006). 16005020 10.1016/j.jtbi.2005.05.020

[b60] Jakobsson L. . Endothelial cells dynamically compete for the tip cell position during angiogenic sprouting. Nature cell biology 12, 943–953 (2010). 20871601 10.1038/ncb2103

