# Supplementary material for: Effects of endothelial cell proliferation and migration rates in a computational model of sprouting angiogenesis
Source: Sci Rep. 2016 Nov 14;6:36992. doi: 10.1038/srep36992 (PMC5107954; doi:10.1038/srep36992)
Supplement: Supplementary Information [file srep36992-s1.pdf]

Effects of endothelial cell proliferation and migration rates in a computational model of sprouting angiogenesis

Kerri-Ann Norton and Aleksander S. Popel
Department of Biomedical Engineering, School of Medicine, Johns Hopkins University
Baltimore, MD 21205

Methods

VEGF Gradients
We examined VEGF gradients in the in silico model to see how gradients would affect cell growth. We examined a linear gradient of VEGF in the x direction with the minimum value being 0.54 ng/ml to the maximum value of 20.5 ng/ml. We choose these values based on in vivo experimental data indicating that VEGF must be greater than 0.5 ng/ml to activate branching angiogenesis[1, 2] and because 20 ng/ml was the value used in our constant VEGF simulations.

Results

VEGF Gradients
We examined VEGF gradients in the in silico model to see how gradients would affect cell growth. We examined a linear gradient of VEGF in the x direction, Supplemental Figure 1. We show three examples, A) with PR of 0.083 1/hr and MR of 10 μ/hr, B) PR of 0.025 1/hr and MR of 4 μ/hr, and C) PR of 0.025 1/hr and MR of 10 μ/hr . We found that, as expected, the growth of the vasculature mostly ran in parallel to the x-axis with branches extending from the sides. This has a similar morphology to skeletal muscle. Interestingly, high PR rates still lead to vascular coverage since the VEGF gradient caused the capillaries to follow the same or similar directions and thus not intersect with one another.

We examined the vascular metrics for these examples and found interesting results. Bifurcation densities at Day 20 were A) 296, B) 128, and C) 216 in 1/mm^3. Vascular length densities at Day 20 were A) 41.7, B) 21.8, and C) 31.1 in mm/mm^3. These values were intermediate compared with the rest of the data. The vascular segment lengths were A) 0.141, B) 0.170, and C) 0.144 mm. These values were higher compared to the rest of the simulations. This is because the VEGF gradient causes more segments to follow the same path before switching directions, thus leading to longer segments. The tortuosity values were A) 1.02, B) 1.01, and C) 1.01 mm/mm. Unsurprisingly, these are very low tortuosity values since most of the vasculature follows straight paths towards the higher VEGF values.

Lastly, the fractal dimension values were A) 1.83, B) 1.76, and C) 1.79. These values were intermediate compared to the rest of the simulations.

The clear difference in the morphologies of tumor vasculature with a VEGF gradient and a constant VEGF value supports the hypothesis that tortuous vasculature in tumors may be due to a constant (and high) value of VEGF within the tumor leading to no clear gradient within the tumor. Outside of the tumor there would presumably be a gradient that would lead the vasculature to the tumor but once the vasculature reached the tumor, there may be no clear gradient leading to tortuous vessels characteristic of tumor vasculature.

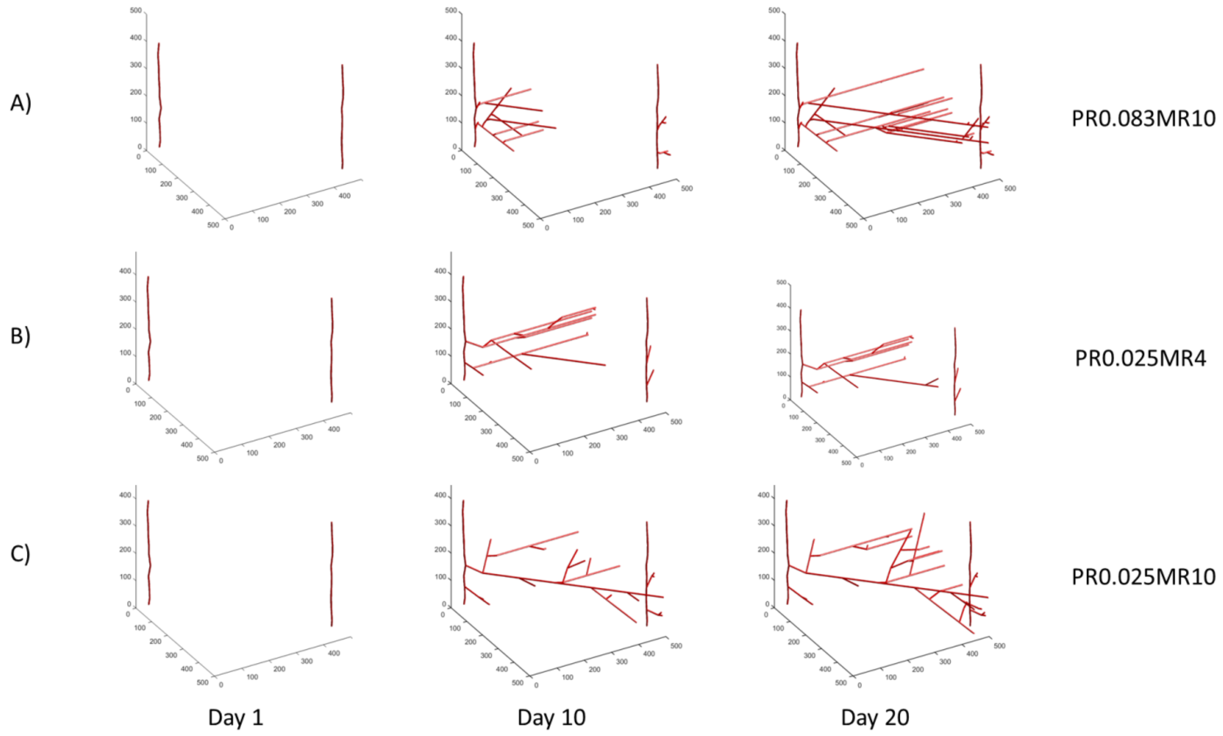

Supplementary Figure 1: Simulations of Vascular Growth with a VEGF Gradient Over Time. A) Example simulation with high proliferation, PR=0.083 1/hr and medium migration, MR = 10 μ/hr. B) Example simulation with medium proliferation rate, PR= 0.025 1/hr and low migration, MR = 4 μ/hr. C) Example with medium proliferation, PR=0.025, and medium migration, MR =10 μ/hr. The vessels all have low tortuosity.

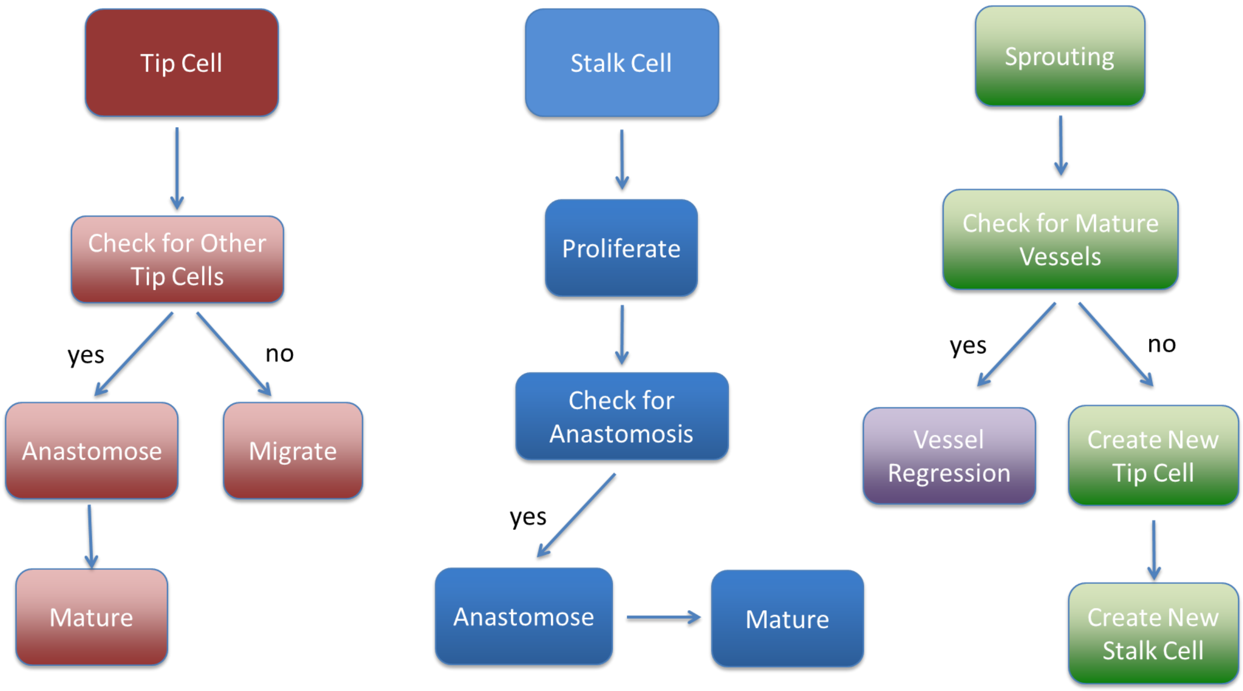

Supplementary Figure 2: Flowchart of the Sprouting Angiogenesis model. First, the model cycles through the capillaries and checks whether the tip cell is near other tip cells. If the check is positive, the tip cell anastomoses with the nearby tip. If it does not anastomose, it migrates, as long as the migration does not cause it to leave the grid. After migrating, it checks whether it has hit another endothelial cell and if so, anastomoses. Next, the adjacent stalk cell is checked whether it can proliferate. If the check is positive, the stalk cell proliferates. Once the cell proliferates, it checks whether it hit another endothelial cell and if so, it anastomoses. After anastomosis the entire capillary becomes mature. Next, the rest of the quiescent phalanx cells are checked to see whether they will sprout. The phalanx cells chosen to sprout start a branch and a new tip cell is formed. The new branch checks whether it is within a certain radius from a mature vessel and if so, it regresses. Once the new tip cell completes its cell cycle, it proliferates and creates a new stalk cell.

Parameter	Value	units
VEGF	20	ng/ml
P_s	6 to 48	hours
σ_s	1	μ
δ	16	μ
d_{base}	6.2	μ/hr
d_{max}	60	μ
σ	0.524	
γ	1	
d_r	50 to 200	μ
e_{max}	30	μ

Supplementary Table 1: Parameter Values in the Computational Model.

1. Hellstrom, M. *et al.* Dll4 signalling through Notch1 regulates formation of tip cells during angiogenesis. *Nature* **445**, 776-780 (2007).
2. Qutub, A.A. & Popel, A.S. Elongation, proliferation & migration differentiate endothelial cell phenotypes and determine capillary sprouting. *BMC Syst Biol* **3**, 13 (2009).
